# Medical waste management training for healthcare managers - a necessity?

**DOI:** 10.1186/2052-336X-11-20

**Published:** 2013-07-16

**Authors:** Aclan Ozder, Bahri Teker, Hasan Huseyin Eker, Selma Altındis, Merve Kocaakman, Oguz Karabay

**Affiliations:** 1 Faculty of Medicine, Department of Family Medicine, Bezmialem Vakif University, Fatih, Istanbul, Turkey; 2 Department of Infectious Diseases, Nisa Hospital, Yenibosna, Istanbul, Turkey; 3 Faculty of Medicine, Department of Public Health, Bezmialem Vakif University, Fatih, Istanbul, Turkey; 4 Faculty of Business, Department of Health Administration, University of Sakarya, Adapazari, Turkey; 5 Vocational High School, University of Yalova, Yalova, Turkey; 6 Faculty of Medicine, Department of Infectious Diseases, University of Sakarya, Adapazari, Turkey

**Keywords:** Healthcare managers, Medical waste training, Medical waste management

## Abstract

**Background:**

This is an interventional study, since a training has been given, performed in order to investigate whether training has significant impact on knowledge levels of healthcare managers (head-nurses, assistant head nurses, hospital managers and deputy managers) regarding bio-medical waste management.

**Methods:**

The study was conducted on 240 volunteers during June – August 2010 in 12 hospitals serving in Istanbul (private, public, university, training-research hospitals and other healthcare institutions). A survey form prepared by the project guidance team was applied to the participants through the internet before and after the training courses. The training program was composed of 40 hours of theory and 16 hours of practice sessions taught by persons known to have expertise in their fields. Methods used in the analysis of the data chi-square and t-tests in dependent groups.

**Results:**

67.5% (162) of participants were female. 42.5% (102) are working in private, and 21.7% in state-owned hospitals. 50.4% are head-nurses, and 18.3% are hospital managers.

A statistically significant difference was found among those who had received medical waste management training (preliminary test and final test) and others who had not (p<0.01). It was observed that information levels of all healthcare managers who had received training on waste management had risen at the completion of that training session.

**Conclusion:**

On the subject of waste management, to have trained healthcare employees who are responsible for the safe disposal of wastes in hospitals is both a necessity for the safety of patients and important for its contribution to the economy of the country.

## Introduction

Wastes that emerge from healthcare institutions are named as hospital wastes, and cause considerable environment pollution. Such wastes represent significant health risks for the hospital employees, patients and the society. Hospital wastes are categorized as household wastes, medical wastes, chemical wastes, infective wastes, chemical wastes and radioactive wastes [1,2].

Of the hospital wastes, 80% are household wastes, 15% are pathological and infective wastes, 3% are chemical and pharmaceutical wastes and 1% are radioactive and cytotoxic wastes [3].

Frequent accidents to the individuals and infection developments in medical institutions generating medical wastes are common occurrences. Severe virus infections such as HIV, Hepatitis B and C are most commonly caused by contaminated wastes that contain incisory-piercing instruments such as hypodermic needles [2,4]. However, since categorizing entire hospital wastes as infected material would increase both financial and labor costs, it is highly important to separate them at the location of their use [5]. Where such wastes are not managed and disposed of by appropriate methods, they constitute serious threats to human health and the environment, and generate an important issue in terms of public health. It is therefore necessary to have wastes separated where they are produced [2].

Collection, transportation and disposal of medical wastes according to world standards were mandated by the publication of “Regulation on the Control of Medical Wastes” in the issue 25883 of the Official Gazette dated 22.07.2005. It is therefore necessary to develop hygienic and economical systems for the disposal of medical wastes where they are regularly collected, categorized and separated at the point of their source (Issue 25883 of the Official Gazette, dated 22.07.2005); (WHO).

According to the official regulation, the Ministry of Environment is responsible for the dispersion of the trainings on waste disposal. Relevant legislation assigns the Head doctor with the responsibility for the organization of the waste management training system in healthcare institutions. However, due to his/her congested agenda, the trainings are usually organized by the Head Nurses, Assistant Head Nurses or Hospital Managers, and subjects taught by head nurses in charge of training or infection-control.

To prevent injury to healthcare employees, patients and the environment by medical wastes, persons responsible for waste management ought to have the requisite knowledge, attitude and behavior [6]. Moreover, it is inevitable to produce an effective management plan in healthcare institutions for the separation of wastes and to have medical wastes controlled and rendered harmless. The realization of these goals primarily necessitates the healthcare managers in hospitals to have sufficient knowledge on the subject. Success in medical waste management may be achieved at in-house training in hospitals instructed by sufficiently knowledgeable managers who have grasped the importance of the subject.

This study was performed in order to investigate whether training has significant impact on knowledge levels of healthcare managers (head-nurses, assistant head nurses, hospital managers and deputy managers) regarding bio-medical waste management.

## Materials and Methods

This study is conducted between June and August 2010. No sampling was used in our study. We included all of the health professionals in all of the hospitals in Istanbul, but volunteers were participated in the study. Managers and executives of all hospitals, having 20 or more beds, under the project of “waste management in healthcare sector”, have been invited. Study is conducted with a total of 240 participants composed of 102 from Private Healthcare Institutions, 65 from Training and Research/University Hospitals, 52 from State-owned Hospitals and 21 from other institutions (Istanbul Provincial Administration, Hemodialysis, Mouth and Dentistry Care Center).

### Partners of the study

This project, supported through the funds from EU and Granada Chamber of Commerce, was realized under the auspices of the Istanbul Local Government by the participation in the project of Yıldız Technical University, Gümüşhane University, Health Administration of Istanbul Province, Environment and Forestry Administration of Istanbul, Health Administration of Gümüşhane, Environment Protection and Recycling of wastes Inc., (İSTAÇ) of Istanbul Metropolitan Municipality, İzmit Waste and Garbage Treatment, Incineration and Recycling Inc. (İZAYDAŞ), Granada University (Spain), Granada Chamber of Commerce (Spain), Athısa Foundation (Spain), Extenda (Spain) and Athisa Group.

### Trainings

Following subjects consistent with Turkish and EU legislation were dwelt on in this project:

• Practical applications of classification, transportation and disposal processes of medical wastes at their source were taught interactively on the internet in Turkey and other countries (Spain, Portugal and Morocco and others).

• A total of 40 instructors, all experts in their fields of expertise, composed of 25 from Turkey and 15 from other countries, gave live lectures on the project, where trainees received 40 hours on the theory and 16 hours on the practical aspects of the project. The participants were able to join the presentations interactively and put in their written, verbal and visual questions or opinions on the subjects under discussion.

• As to practical training, İSTAŞ A.Ş and İZAYDAŞ A.Ş., the companies that perform transportation and disposal of medical wastes and also exemplary hospitals where those topics are deployed were visited, and implementation of medical waste management and applications were observed *in situ*.

### Measurement of the effectiveness of the training

Before the beginning of the research, a survey on the internet was conducted on all healthcare managers to determine the level of information of the healthcare sector on waste management. The survey held before and after the training, consisted of 42 questions on some of the socio-demographic characteristics of healthcare managers and their level of information on collection, transportation and disposal of medical wastes (Table 1). Questionnaire was prepared and applied to a small group (ten people) in order to determine whether questions were understood in the right manner by the participants. Questionnaire was revised according to results and applied to entire group.

**Table 1:** Demographic features of healthcare managers

**Features**		**n**	**%**
**Gender**	Female	162	67,5
	Male	78	32,5
**Institution**	Private Hospital	102	42,5
	Training and Research Hospital-University Hospital	65	27,0
	State Hospital	52	21,7
	Other	21	8,8
**Age**	Below 35	78	32,5
	35-44	99	41,2
	45 and above	63	26,3
**Working period**	Under 5 years	94	39,2
	5-9 years	55	22,9
	10-14 years	41	17,1
	More than 15 years	50	20,8
**Duty**	Head Doctor	12	5,0
	Head Nurse	121	50,4
	Supervisor Nurse	25	10,4
	Hospital Manager	44	18,3
	Supervisor for Medical Waste	23	9,6
	Other	15	6,3
**Total**		**240**	**100,0**

The number of participants was 285 before the application of the survey, and 240 in the course evaluation test held after the research. Individuals who had not participated in the course evaluation test or those who had not answered the questions in their entirety were excluded from the result of the research.

Study had not been given ethical clearance because, it is not necessary for studies not influencing human health directly according to our country’s regulations.

Data collected in the computer before and after the study were evaluated at SPSS 11.5 package program. Chi-Square method was used in the statistical analysis, and the test was implemented in the dependent groups where p<0.05 was taken as statistically significant. Descriptive statistics like percentages and means have also been used in statistical analysis.

## Results

Of 240 participants, 67.5% were female and 32.5 (78) were male of whom 41.2% were between the age range of 35–44. 42.5% (102) of individuals work in private, and 21.7% in public hospitals of whom 50.4% are employed as head nurse, 18.3% as hospital manager, 10.4% as responsible nurse, 9.6% as personnel responsible for medical wastes and 5% as hospital head doctor. Of the participants, 39.2% have declared to have worked for less than 5 years in the institution. Other demographic characteristics of the group are given in (Table 1).

Statistically significant differences were found between the points received by all healthcare managers in the preliminary test and final test (p<0.05) (Table 2). The survey disclosed that the points received by all the healthcare managers were higher in the final test in comparison to the preliminary test. Points received between the preliminary and final tests were, respectively, 31.52±3.47 and 38.36±1.99 for the head doctor, 32.31±4.44 and 38.09±2.90 for head nurses, 31.50±4.25 and 35.84±6.12 for responsible nurses, 29.65±5.33 and 33.21±7.51 for hospital managers, 30.75±7.22 and 37.41±3.14 for personnel responsible for medical wastes, 29.46±3.85 and 35.93±3.84 for others. As a conclusion, it is observed that knowledge level of all healthcare managers has increased as a result of training.

**Table 2:** Comparison of demographic features of healthcare managers due to points from preliminary test and final test

**Variable**		**Preliminary test**	**Final test**	**Statistical analysis**	
		$\bar{X}$ **±S.S**	**X±S.S**	**t**	**p**
**Age**	Below 35 (n:78)	31,08±5,34	36,60±4,37	−9,42	0,000*
	35-44 (n:99)	32,19±3,61	37,35±3,83	−12,42	0,000*
	45 and above (n:63)	31,19±4,97	37,20±5,59	−7,85	0,000*
**Gender**	Female (n:78)	32,13±4,26	37,85±3,00	−7,40	0,000*
	Male (n:162)	30,40±5,08	35,45±6,37	−16,26	0,000*
**Institution**	Private Hospital (n:102)	31,36±4,47	35,93±5,14	−12,16	0,000*
	Training and Research Hospital-University Hospital (n:65)	31,15±5,32	38,16±4,53	−12,16	0,000*
	State Hospital (n:52)	32,48±3,30	37,94±3,19	−8,54	0,000*
	Other (n:21)	31,61 ±5,61	37,04±2,41	−3,84	0,000*
**Duty**	Head Doctor (n:12)	31,52±3,47	38,36±1,99	−8,64	0,000*
	Head Nurse (n:121)	32,31±4,44	38,09±2,90	−13,43	0,000*
	Supervisor Nurse (n:25)	31,50±4,25	35,84±6,12	−5,09	0,000*
	Hospital Manager (n:44)	29,65±5,33	33,21±7,51	−3,55	0,002**
	Supervisor for Medical Waste (n:23)	30,75±7,22	37,41±3,14	−2,85	0,016**
	Other (n:15)	29,46±3,85	35,93±3,84	−7,41	0,000*
**Working period**	Below 5 years (n:94)	32,00±4,30	38,00±2,00	−10,44	0,000*
	5-9 years (n:55)	30,45±5,17	35,70±5,62	−9,62	0,000*
	10-14 years (n:41)	32,40±5,90	38,15±2,29	−5,85	0,000*
	15 years and above (n:50)	32,59±3,18	38,34±4,62	−7,36	0,000*

** p<0.05, * p<0.01.

A ratio of 33.8% of male and 20.1% of female participants have declared to have had no previous training on medical wastes. Whereas, 54.4% of the male and 53.0% of the female of the participants claimed to have received an annual minimum of one training course on the subject. According to gender, the ratio of those having received training on a medical waste management program was statistically higher in females than in males X_female_= 32.13±4.6; X_male_= 30.40±5.08) (p<0.005) (Table 3).

**Table 3:** Comparison of demographic features of healthcare managers according to state of having training in related subject before joining the training programme

**Variables**		**State of having training in related subject before joining the training programme**						**Statistical analysis p value**
		**Never**		**Once**		**At least 3 times**		
		**n**	**%**	**n**	**%**	**n**	**%**	
**Gender**	Male	23	33,8	37	54,4	8	11,8	0,015**
	Female	30	20,1	79	53,0	40	26,8	
**Institution**	Private Hospital	14	15,7	44	49,4	31	34,8	0,012**
	Training and Research Hospital-University Hospital	18	31	33	56,9	7	12,1	
	State Hospital	14	28,6	28	57,1	7	14,3	
	Other	7	33,3	11	52,4	3	14,3	
**Age**	Below 35	17	23,6	34	47,2	21	29,2	0,023**
	35-44	23	26,7	41	47,7	22	25,6	
	45 and above	13	22,0	41	69,5	5	8,5	
**Duty**	Head Doctor	7	28,0	12	48,0	6	24,0	0,084
	Head Nurse	23	20,2	63	55,3	28	24,6	
	Supervisor Nurse	9	23,7	25	65,8	4	10,5	
	Hospital Manager	7	38,9	4	22,2	7	38,9	
	Supervisor for Medical Waste	2	16,7	8	66,7	2	16,7	
	Diğer	5	50,0	4	40,0	1	10,0	
**Working period**	Below 5 years	26	33,3	37	47,4	15	19,2	0,105
	5-9 years	13	27,1	27	56,2	8	16,7	
	10-14 years	4	10,3	22	56,4	13	33,3	
	15 years and above	9	18,8	27	56,2	12	25,0	

** p<0.05.

It was determined that of 15.7% of participant employees in private hospitals, 31% of employees in teaching and research/university hospitals, and 14.0% in public hospitals had received no training whatsoever on the subject of waste management. In comparison, 49.4% of participant employees in private hospitals, 56.9% of employees in teaching and research hospitals and 57.1% in public hospitals have claimed to have attended at least one training course per year on waste management. It was observed that those that had received at least one training course per year in institutions as a whole constituted a majority of the participants (Table 3).

According to the findings, the ratio of employees in private hospitals who attended a minimum of three courses per year in waste management before the program were higher, and that the ratio of female employees having received waste management training were higher than males (Table 3).

When the frequency of waste management trainings of managing healthcare personnel during their function and assignment tenure was compared with those of other participants, no statistically different data were found (p<0.005) (Table 3).

With the exception of hospital managers, approximately half the number of other healthcare managers (personnel responsible for medical waste, head doctor, nurse and head nurse) were observed to have participated in at least one training course on the subject. This ratio in personnel responsible for medical waste and responsible nurses is higher (respectively 66.7% and 65.8%).

As a result of an analysis based on the status of health managers who have received training on the subject, it was found that those who had not received training before and after the preliminary and final tests had scored an average of lower grades that those who had received them (Table 4).

**Table 4:** Comparison of preliminary test and final test points of healthcare managers regarding medical waste training programme

**State of having training regarding medical waste**	**Preliminary test**	**Final test**	**Statistical analyses**	
	**X±S.S**	**X±S.S**	**t**	**p**
Never	30,85±3,88	36,18±5,37	- 6,54	0,000*
Once	31,86±5,28	37,31±4,14	−10,91	0,000*
At least 3 times	32,16±3,42	38,04±2,87	−11,92	0,000*

* p<0.01.

According to the data of the research, 27% of the participants responded to the question “What is the greatest problem encountered in the disposal of medical wastes?” with that sufficient attention was not paid, and auditing was lacking, 20.8% referred to the intensity of works, and 19% claimed the insufficiency of knowledge on the subject. No statistically significant difference among the answers to of the subject of the greatest problem encountered in the disposal of medical wastes were found as to the gender of healthcare managers, their age groups, duration of their tenure and functions (Table 5).

**Table 5:** Comparison of demographic features of healthcare managers regarding problems in disposal of medical wastes

**Variable**		**The most important problems in disposal of medical wastes**										
		**Insufficiency of knowledge**		**Intensity of work**		**Being out of reach of medical waste bags**		**Lack of auditing**		**Other**		
		**n**	**%**	**n**	**%**	**n**	**%**	**n**	**%**	**n**	**%**	**p value**
**Gender**	Male	15	19,2	13	16,7	20	25,6	23	29,5	7	9,0	0,175
	Female	31	19,1	37	22,8	25	15,4	42	25,9	27	16,7	
**Institution**	Private Hospital	20	19,6	21	20,6	20	19,6	25	24,5	16	15,7	0,96
	Training and Research Hospital-University Hospital	12	18,5	11	16,9	13	20,0	21	32,3	8	12,3	
	State Hospital	9	17,3	12	23,1	8	15,4	16	30,8	7	13,5	
	Other	5	23,8	6	28,6	4	19,0	3	14,3	3	14,3	
**Age**	Below 35	18	23,1	18	23,1	12	15,4	24	30,8	6	7,7	0,355
	35-44	14	14,1	18	18,2	23	23,2	26	26,3	18	18,2	
	45 and above	14	22,2	14	22,2	10	15,9	15	23,8	10	15,9	
**Working period**	Below 5 years	20	21,7	15	16,3	24	26,1	23	25,0	10	10,9	0,395
	5-9 years	11	20,4	12	22,2	9	16,7	17	31,5	5	9,3	
	10-14 years	7	17,5	11	27,5	5	12,5	9	22,5	8	20,0	
	15 years and above	7	14,3	12	24,5	6	12,2	14	28,6	10	20,4	
**Duty**	Head Doctor	7	28,0	5	20,0	3	12,0	8	32,0	2	8,0	0,180
	Head Nurse	21	17,4	28	23,1	17	14,0	30	24,8	25	20,7	
	Supervisor Nurse	7	15,9	8	18,2	13	29,5	11	25,0	5	11,4	
	Hospital Manager	4	17,4	4	17,4	7	30,4	6	26,1	2	8,7	
	Supervisor for Medical Waste	1	8,3	4	33,3	2	16,7	5	41,7	0	0	
	Other	6	40,0	1	6,7	3	20,0	5	33,3	0	0	

Suggestions of 70% of the participants for the solution of the problem were on the necessity of effective training and audit, and 30% of them proposed to have the personnel employed in waste disposal and general cleaning to be issued certificates. They have also stated the greatest problem encountered by healthcare personnel on the subject (in all health institutions (private, training and research/university and public) was the lack of auditing. According to the research results, health managers in all age groups cited the primary problem on the subject as the inadequacy of emphasis.

## Discussion

All wastes produced by the health institutions constitute a health risk for the hospital employees, patients and the environment. If these wastes are not collected, stored and disposed of by appropriate methods, they will emerge as severe environment and public health problems. Healthcare managers bear the primary responsibility for the collection and storage of such wastes in health institutions. Information levels and awareness of health managers on the subject are important in waste management.

A research in the literature indicated that the majority (69.9%) of health employees had received training of the subject of medical wastes [6]. Also, a research which has been made to evaluate the level of information of hospital health personnel on waste management revealed that, in general, among the professional health employees, 62.1% of medical doctors, 54.5% of nurses and 47.6% of laboratory technicians had more information on the subject [7]. In a similar manner, a study showed that medical doctors, nurses and laboratory technicians were better informed on waste disposal than the cleaning personnel [8]. Also, a study by Suwarna and Ramesh in 2012 demonstrated that doctors and nurses that had a higher level of information than other hospital personnel [9].

A another survey for the determination of information levels, the factors that affected the information levels on medical wastes, and organizing their training programs conducted among 453 hospital healthcare personnel disclosed that information of 43.5% of the participants averaged at medium level. In the same study, specialist doctors, the interns and nurses were found to be better informed than the other hospital personnel, but the cleaning employees had a significant information deficiency on the subject [1].

Lakshmi and Kumar conducted an analysis among the healthcare workers on the awareness of biomedical waste management. In that study, researchers report in their findings as having detected an information and awareness deficiency among the hospital employees as to the legislation associated with biomedical waste management. In this study, performed on qualified hospital employees also indicates that a knowledge and awareness deficiency exist among the qualified hospital personnel about the legislation on biomedical waste management [10].

The result of our study too is consistent with the conclusions of a multiple number of research papers delving into the information level on medical waste of health workers waste [1,6,9,10].

The study revealed that all healthcare managers had better scores in the final test than the preliminary test. According to the findings, the information levels of all healthcare managers had improved after the training, which indicated the effectiveness of the training supplied supplied to the study group. As cited by Hasçuhadar *et al.*, the amount of waste produced in hospitals increases day by day in proportion to the services provided. To eliminate the danger posed by this growing amount to human and environment health, all healthcare personnel should be supplied with “Hospital Medical Waste Plan”, and regularly given trainings on where each type of waste should be deposited [1].

In a study aimed at achieving waste control in a new hospital, a person responsible for waste control was assigned to a post and all personnel were individually subjected to a regular and continual in-house training. The hospital head nurse and the infection control nurse were sent to external training and certificated. As a result, the number of wrong applications in the collection of household and medical wastes were reduced to 5, where only one case of personnel and environment infection occurred, and there were no injuries due to cutting-piercing tools. Moreover, the amount of medical waste generated in the hospital was reduced, thus reducing hospital expenditures [11].

Among the subjects of our study, 15.7% of the hospital employees in private hospitals, 31.0% in teaching and research/university hospitals and 14.0% of the public hospitals declared to have had no training whatsoever on medical wastes.

When the averages between the preliminary test and final test are examined among the healthcare managers who have attained one or more training courses, it will be seen that the averages are raised at the end of each training (Table 4). This situation leads us to think that the tests highlight a deficiency in the information level of the managers that should be repaired with periodic training programs. It may therefore be proposed to draw an effective waste management plan in hospitals, and have that plan continually implemented by periodical training, so as to repair the information deficiency of healthcare managers.

Private hospitals providing a minimum of three training sessions each year to their employees, an average higher than other healthcare institutions is indication of their organizing more frequent waste management training courses. This, in turn, may be a sign of a higher importance attached to medical wastes in those institutions.

From the answers to the question “How many times have you attended medical waste training programs?” put to the personnel participating in the study, a significant statistical difference was found between the attendance status of the subject and the points he/she had scored in the preliminary and final tests (p<0,01). Health sector managers who had attended previous training programs, having received higher points than those who had not is a sign of the continuity and necessity of training.

In a study carried out by Hasçuhadar *et al.* the reasons given by the subjects to the question “What are the problems you encounter on the matter of medical wastes?” were “Intensity of my work” (32.9%) and “Supervision deficiency in the department” (29.4%) [1]. Similar responses were received in our study, too. In our research, we found no significant statistical difference between the answers to the question inquiring about the most important issue in medical waste disposal, and the gender, age group, duration of employment and position of the healthcare managers (p>0,05). Similar responses from healthcare managers without gender, age group, duration of employment and position difference between them is significant. Approximately 70% of the participants proposed effective training and supervision to solve the problem. The other 30% suggested to have the medical waste personnel and cleaning employees certificated. To organize and implement a standardized training program for all employees regardless of their position in all healthcare institutions would play an important role in the solution of this issue. Also, supervisions by healthcare managers and specialist firms would provide an important contribution of the same issue.

## Conclusion

This study has determined that training programs on the subject of waste management in the health sector has a significant effect in increasing the information level of the healthcare personnel. The most important problem in waste management was underscored as not having sufficient training and supervision. Among the solution propositions, set alongside effective training and supervision, is having the cleaning personnel subjected to a standard training program, and them certificated for proficiency.

Preparation of an effective waste management plan in hospitals, application of a periodic training program at all levels, and supervision by institutions specialized in that field would provide an important contribution to the waste management.

## Competing interests

The authors declare that they have no competing interests.

## Authors’ contributions

AO conceived of the study, and participated in its design and coordination and helped to draft the manuscript. BT carried out data collection. HHE performed the data analysis. SA helped to draft the manuscript. MK participated in the design of the study. OK made critical revisions of manuscript for important intellectual content. All authors read and approved the final manuscript.
